# Reduced cortical microvascular oxygenation in multiple sclerosis: a blinded, case-controlled study using a novel quantitative near-infrared spectroscopy method

**DOI:** 10.1038/srep16477

**Published:** 2015-11-13

**Authors:** Runze Yang, Jeff F. Dunn

**Affiliations:** 1 Department of Radiology, Cumming School of Medicine, University of Calgary; 2 Hotchkiss Brain Institute, Cumming School of Medicine, University of Calgary; 3 Department of Clinical Neurosciences, Cumming School of Medicine, University of Calgary.

## Abstract

Hypoxia (low oxygen) is associated with many brain disorders as well as inflammation, but the lack of widely available technology has limited our ability to study hypoxia in human brain. Multiple sclerosis (MS) is a poorly understood neurological disease with a significant inflammatory component which may cause hypoxia. We hypothesized that if hypoxia were to occur, there should be reduced microvascular hemoglobin saturation (S_t_O_2_). In this study, we aimed to determine if reduced S_t_O_2_ can be detected in MS using frequency domain near-infrared spectroscopy (fdNIRS). We measured fdNIRS data in cortex and assessed disability of 3 clinical isolated syndrome (CIS), 72 MS patients and 12 controls. Control S_t_O_2_ was 63.5 ± 3% (mean ± SD). In MS patients, 42% of S_t_O_2_ values were more than 2 × SD lower than the control mean. There was a significant relationship between S_t_O_2_ and clinical disability. A reduced microvascular S_t_O_2_ is supportive (although not conclusive) that there may be hypoxic regions in MS brain. This is the first study showing how quantitative NIRS can be used to detect reduced S_t_O_2_ in patients with MS, opening the door to understanding how microvascular oxygenation impacts neurological conditions.

Hypoxia has been implicated in a range of neurological disorders including stroke, Alzheimer’s^1^, chronic obstructive pulmonary disease (COPD)^2^, birth asphyxia, high altitude cerebral edema, microvascular disease^3^, and multiple sclerosis^4^. Hypoxia will also modulate the inflammatory response in brain, which could impact progression of inflammation related diseases including MS. It has been difficult to study hypoxia in the brain due to measurement limitations but new technology such as frequency domain near-infrared spectroscopy (fdNIRS) systems can be applied to quantify microvascular hemoglobin saturation (S_t_O_2_) as a potential marker of hypoxia in MS. We undertook this study to determine if reduced S_t_O_2_ is a common component of the pathophysiology of MS.

Although MS has been considered a white matter disease, it is now clear that there is diffuse gray matter involvement^5^. In the grey matter (GM), there is both diffuse and focal demyelination and axonal loss^5^. There is evidence that both metabolism and blood flow may be impaired^6^. These data support the concept that metabolic and histological damage occurs throughout the GM. For this reason we are not looking for S_t_O_2_ in plaques, but looking at average cortical gray matter.

Hypoxia can have profound effects on the metabolic pathways implicated in MS as well as on basic CNS function. Hypoxia can exacerbate inflammation, through common regulating signals involved in both the inflammatory and hypoxia responses^7^. The most well studied signal linking hypoxia and inflammation is through the prolyl hydroxylases (PHD). Prolyl hydroxylases act as an oxygen sensor, but it will also stimulate the NF-κB pathway and promote inflammation under hypoxic conditions. Hypoxia and inflammation are intricately linked, making it important to determine if hypoxia exists in MS.

Although there is evidence for hypoxia, it is largely correlative or circumstantial. Type IV plaques have a hypoxic/ischemic appearance and the hypoxia modulator HIF is up-regulated in patients^8^. A direct measurement of partial pressure of oxygen (pO_2_) indicated hypoxia existed in the spinal cord of an animal model of MS^9^. We are not aware of any direct measurements in patients showing the presence of hypoxia in GM.

We undertook this study due to need for greater understanding of GM involvement. Abnormally low S_t_O_2_ in GM would support the hypothesis of GM involvement and provide a potential biomarker. In addition, it would support the hypothesis that hypoxia may play a role in the pathophysiology of MS. We applied fdNIRS which can be used for absolute quantification of oxyhemoglobin (HbO) and deoxyhemoglobin (HHb)^10^ while most commercially available NIRS systems only detect changes in concentration. By quantifying the absolute values for HbO and HHb using fdNIRS^10^,^11^, we are able to calculate S_t_O_2_.

In this study we applied fdNIRS to quantify S_t_O_2_ in a cohort of MS patients. These data are the first to show, both as a population and on an individual patient basis, that there is significant hypoxia in many MS patients.

## Results

Patient characteristics are summarized in Table 1. There were no significant differences in age between controls, relapsing remitting (RRMS), secondary progressive (SPMS), and primary progressive MS (PPMS) patients.

**Figure 1:**
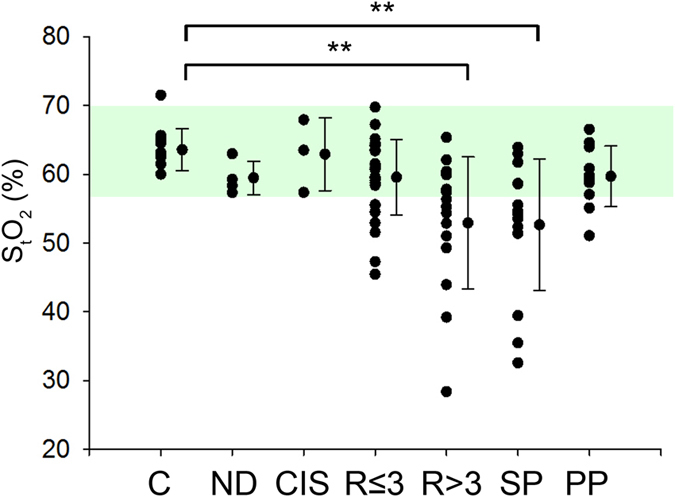
fdNIRS measurement of brain oxygenation shows hypoxia in patients with MS. S_t_O_2_ from individual patients from different MS patient sub-types showing the distribution of hypoxia. Each dot represents one subject. Beside the subject data are the combined mean ± SD. Green shaded area represents 2 × SD around the control mean. All points below the shading are 2 × SD below the controls (significantly hypoxic). C - control; ND - no disease (patients who were referred to the MS clinic but did not have MS/CIS); CIS - clinically isolated syndrome; R ≤ 3 - RRMS patients with EDSS less than or equal to three; R > 3 - RRMS patients with EDSS greater than three; SP - secondary progressive; PP - primary progressive **p < 0.01.

S_t_O_2_ was significantly decreased in SPMS patients compared with controls (p < 0.01) (Fig. 1 and Table 2). As a group, S_t_O_2_ in RRMS patients was not significantly different from controls. To investigate whether severity may relate to oxygenation, we divided the RRMS patients into two groups, with expanded disability status scale (EDSS) greater than, or less than 3. An EDSS score of 3 was used because the time to EDSS 3 is often used to judge aggressiveness of the disease course. RRMS patients with EDSS > 3 had significantly reduced S_t_O_2_ compared to controls, while there was no difference in S_t_O_2_ between controls and RRMS patients with EDSS <= 3. Figure 1 shows the S_t_O_2_ for the patient sub-groups.

The S_t_O_2_ from PPMS patients were not significantly different from controls or the two patient groups RRMS and SPMS. However, it is worthy to note that the 95% CI of the PPMS group had very little overlap with the other patient groups (49–58 for RRMS, 47–58 for SPMS vs. 57–62 for PPMS) and so future work may show that S_t_O_2_ of PPMS is more similar to controls than other patients.

To comment on whether individual patients showed a reduction in S_t_O_2_, we used threshold method to detect significance. A S_t_O_2_ threshold of 2 standard deviations (SD) or more below that of the mean for control subjects was used. By this criteria, there was reduced S_t_O_2_ in sixteen RRMS (36%), ten SPMS (67%) and three PPMS (23%) patients for a total of 42% of all the patients.

**Figure 2:**
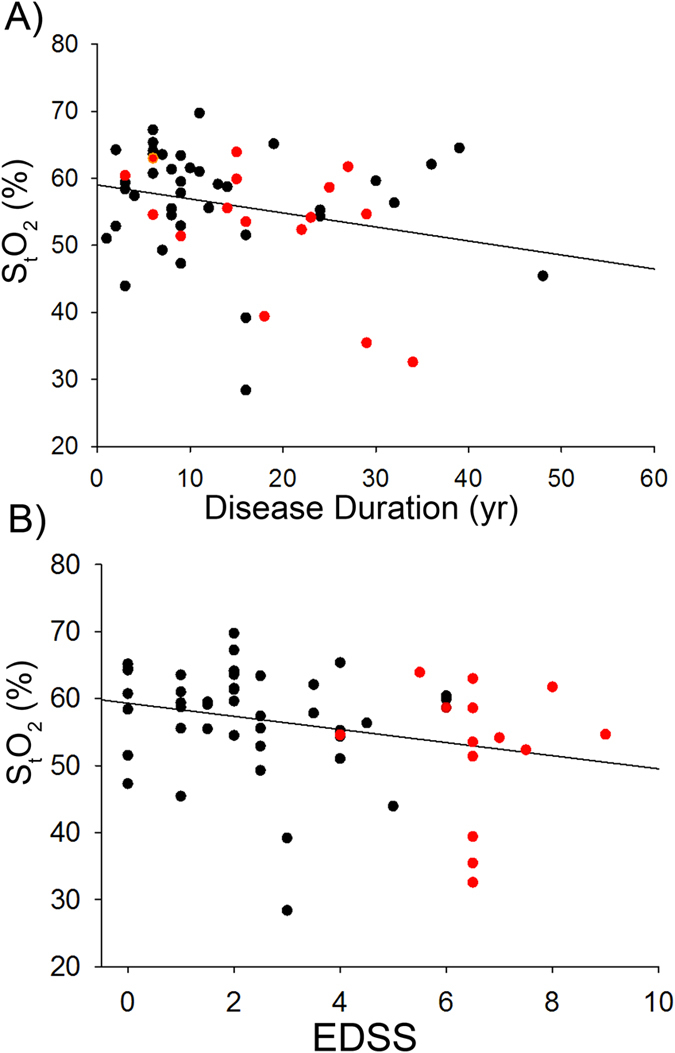
Relationship between S_t_O_2_ and disability. (**A**) Relationship between S_t_O_2_ and disease duration. Red dots denote SPMS patients while black dots denote RRMS patients. There was a significant negative relationship between disease duration and S_t_O_2_ in RRMS + SPMS patients (p < 0.05, Pearson correlation, r = −0.262). (**B**) There was a significant negative correlation between S_t_O_2_ and EDSS scores (p < 0.05, Spearman’s rank test, r = −0.364).

There is a narrow range of EDSS in the RRMS and SPMS group, making correlational analysis for individual groups difficult. Since the majority of RRMS patients will transition into the SPMS stage, and that significant inflammation is present in both groups, we combined the RRMS and SPMS data into one group and carried out an analysis to determine the relationship between S_t_O_2_ and either disease duration or EDSS (Fig. 2A,B). We found a significant negative correlation between S_t_O_2_ and disease duration (Fig. 2A, Pearson’s correlation) as well as between S_t_O_2_ and EDSS (Fig. 2B, Spearman’s correlation used as EDSS is interval data). Multiple linear regression analysis also indicated that lower S_t_O_2_ was associated with clinical measures of disability (Table 3). The clinical measures of disability in the MS subgroups are summarized in Table 4.

**Figure 3:**
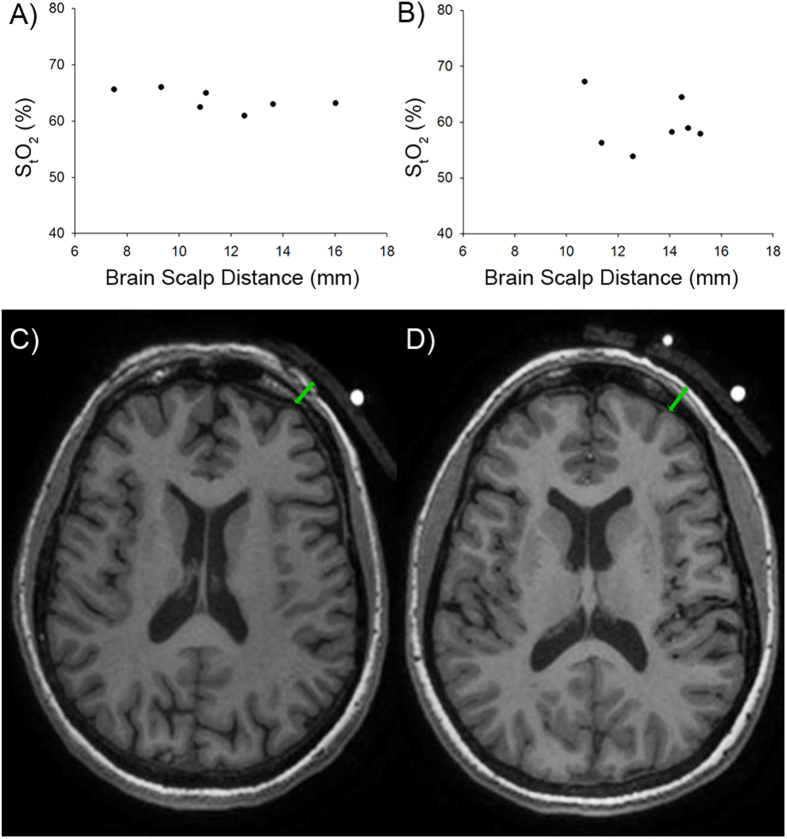
Combined MRI/NIRS measurement. There was no significant relationship between S_t_O_2_ and brain scalp distance in controls (**A**) and MS patients (**B**). Representative images of a control and MS patient are shown in panel C and D, respectively. Bright dot represents the location of the Vitamin E capsule placed besides the source and detector.

In a second study, we undertook concurrent MRI/NIRS measurements (7 controls and 7 MS patients). We found that the RRMS patients (n = 4) had significantly reduced S_t_O_2_ compared to controls (n = 7) (65 ± 5 controls vs 56 ± 2 RRMS, mean ± S.D., t-test, p < 0.05). Even though S_t_O_2_ was significantly lower in the MS subjects, there was no significant difference in brain scalp distance (as a marker of atrophy) (1.15 ± 0.28 cm controls vs 1.33 ± 0.18 cm RRMS). We also found no significant relationship between the brain scalp distance and S_t_O_2_ in either the control or MS groups (Fig. 3A,B).

## Discussion

The microvascular saturation (S_t_O_2_) in the control population was comparable to a previous study using similar NIRS equipment^11^ (64 ± 3% in our study vs. 63 ± 5% mean ± S.D), indicating the reproducibility of the measurements.

The fact that S_t_O_2_ is reduced in SPMS and high disability RRMS patients provides strong evidence for abnormal physiology in GM. It is supportive, although not conclusive, that some level of hypoxia exists. This reduction in S_t_O_2_ must be relatively diffuse as it is unlikely that, through random chance, we measured over an MS plaque in such a large number of patients. There was a significant correlation between S_t_O_2_ and several of the clinical measures of motor disability (Table 3), disease duration and EDSS (Fig. 2). The fact that only the high disability RRMS patients were significantly different from controls suggests that reduced S_t_O_2_ may relate to disability. As S_t_O_2_ was measured in the frontal cortex, one might expect the cognitive test to show a strong correlation with S_t_O_2_. This was not observed, but there is a relation with motor tests, supporting the view that the reduction in microvascular oxygenation is likely to be distributed through gray matter. More sensitive cognitive testing will need to be undertaken as a future study.

Examining the patients on an individual basis, using 2× SD below the control mean as a criterion, we detected patients with significantly reduced S_t_O_2_ in all groups (Fig. 1). We believe this to be an important finding, as the biomarker of S_t_O_2_ identifies a new subgroup of MS patients with reduced S_t_O_2_ that cross standard classification schemes.

We believe reduced oxygenation in the brain is not the primary cause of MS, given that not all patients show reduced S_t_O_2_ and the severity of the reduction in S_t_O_2_ is relatively mild in many subjects. A reduced S_t_O_2_ may reflect a change in the pattern of supply and demand of oxygen delivery. Supply could be altered if neurovascular coupling is impaired, meaning that for a given increase in energy demand, there is a blunted flow response. Such would be expected if there is endothelial cell damage caused by inflammation. The demand may also change. With the “inside out” hypothesis of MS^12^, the initial trigger could be mitochondrial damage followed by inflammation and white matter loss. There would be a time-course of change in oxygen demand, as demyelinated axons require more energy to transmit an action potential. Another cause of increased oxygen uptake would be increased infiltration of inflammatory cells and increased activation of microglia^13^.

An interesting link between these scenarios is inflammation. Reduced S_t_O_2_ may reflect the cumulative effects of inflammation. As such, reduced S_t_O_2_ may be a sensitive biomarker of level of inflammation and the efficacy of anti-inflammatory treatments. Differences in S_t_O_2_ between the different MS subtypes would support the hypothesis that reduced microvascular oxygenation is related to inflammation. In RRMS patients, there is significant inflammation in the brain. This is seen by the presence of gadolinium enhancing lesions on MRI. In SPMS patients, although the frequency of focal inflammatory lesions is significantly reduced, diffuse inflammation is present, demonstrated using PET and via post-mortem examinations^14^,^15^. On the other hand, although inflammation also exists in PPMS patients, the magnitude of diffuse inflammation is significantly less compared to SPMS^14^.

Animal model work supports a link between inflammation and the presence of hypoxia. Injection of lipopolysaccharide (LPS) into rats results in a model of septic shock^16^. This model shows reduced cortical PO_2_^17^. In addition, spinal cord PO_2_ measurements taken from a rat inflammatory demyelinating model of MS showed that hypoxia coincided with inflammation^9^. Therefore, it is possible that S_t_O_2_ difference between RRMS + SPMS and controls is due to the magnitude of inflammation.

However, using T_2_ to estimate the venous saturation, two studies^18^,^19^ showed that RRMS patients exhibited higher venous oxy-hemoglobin saturation in the lower superior sagittal sinus compared to controls^18^. This is inconsistent with studies using an animal model of MS, the EAE mouse, where they found increased deoxyhemoglobin in the draining veins^20^. Spinal cord PO_2_ was reported to be low (hypoxic) in a rat EAE model of MS^9^. Thus, although these T_2_ data are interesting, it may be too large a jump to assume the brain parenchyma is hyperoxic. For instance, it could also be that there is some arterial-venous shunting in the brain of MS patients, resulting in an overall higher saturation in large veins.

Several studies provide indirect evidence for hypoxia in MS, showing reduced blood flow and presence of hypoxia resembling plaques in patients with MS^8^. Two previous PET studies did not find any significant differences in the oxygen extraction fraction (OEF), a parameter indicative of brain oxygenation, between RRMS patients and controls^6^,^21^. This is consistent with our findings, since RRMS patients in our study did not exhibit reduced S_t_O_2_ when data from all RRMS patients were combined.

The existence of hypoxia in a subgroup of MS may explain some of the variance in treatment response. Hypoxia may exacerbate inflammation^22^, so it is possible that patients with hypoxia may have an altered anti-inflammatory treatment response. Our results may also provide an explanation for the inconsistent findings looking at the efficacy of hyperbaric oxygen therapies (HbOT) in MS. Some studies found significant improvements in clinical scores after HbOT^23^,^24^, while most concluded that there were no significant benefits^25^,^26^. We showed that reduced S_t_O_2_ is present in a subset of MS patients. In patients whose brains are hypoxic, it is possible that re-oxygenating the brain will have clinical benefits. However, in patients whose brain oxygen levels appear normal, it is unlikely that HbOT will produce significant benefits.

NIRS has several advantages compared to other methods of measuring blood oxygenation such as PET or MRI. Unlike PET which uses expensive radioactive isotopes, NIRS uses low energy light to obtain oxy and deoxyhemoglobin concentrations, making it less invasive which allows for frequent and repeated measurements to be made. The ability to directly measure hemoglobin concentrations is advantageous compared to MRI, which indirectly estimates the oxyhemoglobin saturation of large vessels by measuring the (assumed to be deoxyhemoglobin) difference in susceptibility between the outside and inside of the vessel. This works in large vessels but is difficult to apply to the microvasculature. In addition, NIRS systems are portable. Lastly, most NIRS measurements take no more than 5 minutes to complete, which is significantly faster than PET or MRI. Given that NIRS is simple to operate, measurements can be made in an examination room during routine visits.

The major limitation associated with fdNIRS studies is the partial volume effect. A significant portion of the NIRS signal is passing through the scalp and skull before reaching the brain, so the fdNIRS signal is contaminated by the scalp and skull^27^. This would mean that brain atrophy may influence the results, since there will be a change in distance from the optical fibers to the brain.

We believe the impact of brain atrophy on our conclusion is minimal. In the subjects who underwent concurrent NIRS with MRI, we found no significant difference in brain to scalp distance between controls and MS, but observed a difference in mean S_t_O_2_ between controls and RRMS patients. In addition, there was no significant relationship between scalp distance and measured S_t_O_2_ in controls as well as patients (Fig. 3). This shows that reduction of S_t_O_2_ is independent of scalp-brain distance. Furthermore, we showed that reduced S_t_O_2_ in RRMS can occur in patients without brain atrophy.

In MS, total parenchyma fraction has been reported to decrease by approximately 0.7% per year^28^. In our RRMS + SPMS group that showed reduced S_t_O_2_, the mean disease duration is 10 years, suggesting that there will be a 7% decrease in parenchyma fraction. Making a rough assumption that the brain is a sphere, this would result in a 1.9% decline in radius, which is small when considering the total light path.

In addition, brain atrophy would bias the signal towards the S_t_O_2_ of extracerebral tissue, which is not expected to change in patients with MS. It has been shown that there is no significant difference in S_t_O_2_ between the extracerebral tissue (measured using source detector separation of 0.8 cm to 1.8 cm) and cerebral tissue (measured using source detector separation of 2.8 to 3.8 cm)^29^. Therefore, brain atrophy should bias the data towards a high S_t_O_2_. If brain atrophy is impacting our results, it is causing us to underestimate the reduction in S_t_O_2_ and it will not significantly alter our conclusion.

We show that there is reduced cortical microvascular oxygenation (reduced S_t_O_2_) in a significant number of MS patients. This subtype of MS crosses standard classification schemes. This is consistent with the presence of hypoxia in MS. We show that the fdNIRS method is applicable to study MS and other neurological disorders where hypoxia is part of the pathophysiology. A measurement of S_t_O_2_ may provide a sensitive, early response biomarker for treatment response in multiple sclerosis.

## Materials and Methods

### Subjects

Ethics were approved by the Conjoint Health and Research Board at the University of Calgary. The study method was carried out in accordance with the approved guidelines. All subjects were over 18 and underwent informed consent. We recruited 72 MS patients and 3 clinically isolated syndrome (CIS, patients who had only 1 episode of MS attack) patients from the Multiple Sclerosis Clinic at the Foothills Medical Center. Patient demographics are summarized in Table 1. There were 44 RRMS patients. 18/44 of these RRMS patients had EDSS score >3 with a mean age of 51 ± 9 (mean ± S.D.). The mean age for RRMS patients with EDSS ≤3 was 41 ± 11. There were 15 SPMS, 13 PPMS, 12 control subjects, as well as 4 patients referred to the MS clinic who were later diagnosed to not have MS. In addition, we recruited additional 7 MS patients (4 RRMS, 1 SPMS, 2 PPMS) and 7 controls and underwent concurrent NIRS/MRI measurements.

### NIRS System

Most commercially available NIRS systems can only detect changes in concentration. By quantifying the absolute values for HbO and HHb using fdNIRS^10^, we are able to calculate microvascular tissue oxyhemoglobin saturation (S_t_O_2_), an indicator of the oxygenation status of the brain. This allows us to compare NIRS measurements between different groups. An ISS OxiplexTS was used for fdNIRS. The semi-infinite solution to photon diffusion equation was used to calculate hemoglobin concentrations, with water concentrations assumed to be 75%. This system measures optical attenuation while simultaneously estimating path length using a frequency domain method. The estimation of the tissue absorption coefficients at multiple wavelengths enables the oxy and deoxyhemoglobin concentration to be calculated using the Beer Lambert Law. The S_t_O_2_ is calculated as [HbO]/([HbO] + [HHb]).

The probe consists of one detector and four fiber optic sources with a separation of 2·0 to 3·5 cm from the detector. The NIRS probe emits light at 690 and 830 nm into the tissue, with an amplitude modulation frequency of 110 MHz.

### Measurement Protocol

Measurements were made during routine clinical visits. All participants sat in a soft comfortable chair and were asked to relax. The fdNIRS system was warmed up for 10 minutes prior to data collection and calibrated with a phantom calibration block with known absorption and scattering coefficients. The calibration was checked using another phantom with a different absorption and scattering coefficient (check block). The calibration was accepted if the measured absorption and scattering values were within 0.01 cm^−1^ and 0.1 cm^−1^ of the absolute values for the check block.

NIRS data were collected at a rate of 2 Hz for approximately 1 minute in the left and right frontal lobe while the operator manually held the NIRS probe to the forehead. Probes were positioned approximately 2 cm above the eyebrow. The probe edge was 1.5 cm from midline of the forehead making the center of the source/detectors ~4 cm from the midline (Fig. 1).

Measurements were checked to make sure they obey the semi-infinite solution of the photon diffusion equation^10^. This was done by determining that a plot of source detector distance vs. either signal amplitude or phase was linear. An R^2^ value greater than 0.95 was considered acceptable. The probe placements were adjusted until the linear criterion met.

### Clinical Measures of Disability

Patients performed a timed 25 foot^30^ walk twice (results averaged) with the aid of a walker, if necessary. Patients who were non-ambulatory did not perform this test. Two trials of the 9 hole peg test using the dominant, as well as the non-dominant hand, were conducted (results were averaged). The 9 hole peg test^31^ involved placing 9 pegs into 9 slots in a standardized peg board. The time required to insert all 9 pegs into their respective slots was measured. One trial of the symbol digit modalities test (SDMT) was conducted. Using a reference key, subjects were given 90 seconds to match specific numbers with a given geometric figure. The total number of pairs matched and the number of correctly matched pairs were recorded. EDSS scores of the patients were also determined on the day of examination.

### Data Analysis

Data points (120) were averaged from the left and right side of the forehead. The mean S_t_O_2_ was compared between the left and right side using a paired t-test. As there was no significant difference, the left and right side mean data were averaged for each individual.

Analysis of covariance (ANCOVA) with age as a covariate followed by the Tukey’s post-hoc test was done on each fdNIRS parameter to determine whether there were significant differences between controls, CIS patients, RRMS patients, SPMS patients, and PPMS patients. Spearmans correlation was done between S_t_O_2_ and EDSS. A multiple regression was carried out between S_t_O_2_, each score of clinical disability while controlling for age.

### Concurrent MRI and NIRS protocol

MR imaging was performed using a 3T GE Discovery MR750 system with a 12 channeled head coil. Axial anatomical scans were collected using a spoiled recalled gradient acquisition (TR = 8.16 ms, TE = 3.18 ms, Flip angle = 12^o^, Voxel size = 1 mm × 1 mm x 1 mm). For NIRS measurements in the MRI, we worked with ISS OxiplexTS and developed an MRI compatible probe with 4 sources and 1 detector (source detector distance 2.0 cm–3.5 cm). The custom built probe was placed on the left forehead of subjects and secured with Velcro straps and data was collected for the duration of the scan. Vitamin E capsules were placed besides the source and detector to indicate the location of NIRS measurement on the MRI. Linearity between source detector distance and AC/Phase was checked to ensure the quality of the data. 20 slices of MRI images containing the Vitamin E capsule were used to calculate the distance from the brain and scalp.

## Additional Information

**How to cite this article**: Yang, R. and Dunn, J. F. Reduced cortical microvascular oxygenation in multiple sclerosis: a blinded, case-controlled study using a novel quantitative near-infrared spectroscopy method. *Sci. Rep.* **5**, 16477; doi: 10.1038/srep16477 (2015).

## Figures and Tables

**Table 1:** Patient Characteristics.

	Control	RRMS	SPMS	PPMS
n	12	44	15	13
Gender M:F	3:9	10:34	3:13	6:7
Age	50 (8)	43 (11)	55 (6)	53 (8)
Disease Duration (years)	N/A	11 (11)	9 (7)	20 (9)
EDSS	N/A	2·2 (1·6)	6·2 (1·8)	4·7 (2)

Data are presented as mean (SD).

**Table 2:** NIRS parameters for each patient group.

Patient Type	S_t_O_2_ (%)	tHb (μM)	HbO(μM)	HHb(μM)
Control	63·5 (3)	45·3 (7)	28·1 (4)	17·2 (4)
RRMS EDSS = < 3	59·6 (6)	47·1 (11)	28·1 (7)	19·0 (5)
RRMS EDSS >3	52·9 (9)*	41·6 (7)	23·2 (4)	18·4 (4)
SPMS	52·6 (9)*	42·1 (8)	22·4 (7)	19·7 (4)
PPMS	59·4 (4)	44·2 (10)	26·1 (6)	18·1 (5)

tHb = total hemoglobin; HbO = oxyhemoglobin; HHb = deoxyhemoglobin. Data are presented as mean (S.D). There were significant differences in HbO between the five groups. A post-hoc tukey’s test showed no significant differences in HbO between any pair of subjects. *significantly different from control, p < 0·05.

**Table 3:** Relationship between S_t_O_2_ and measures of clinical disability.

Clinical Test	p value(S_t_O_2_)	pvalue(age)	pvalue(S_t_O_2_, age)	Slope(+/−)	rvalue
9 Hole Peg - ND	0·018	0·291	0·018	−	0·414
25 Ft Walk	0·034	0·155	0·016	−	0·423
9 Hole Peg - D	0·182	0·041	0·017	−	0·377
SDMT	0·405	0·132	0·134	+	0·265

Results of a multiple regression analysis with the clinical test results as the dependent variable, and age as well as S_t_O_2_ as the independent variable. The slope direction of the impact of S_t_O_2_ on clinical test outcomes is also reported. P-value for S_t_O_2_ and P-value for age shows whether or not that particular variable is significantly associated with the testing scores. P-value for S_t_O_2_ plus age shows whether or not entire regression (taking into account both S_t_O_2_ and age) is associated with the testing results.

**Table 4:** Clinical assessment results of patient groups (mean (SD)).

Clinical Test	RRMSEDSS = < 3	RRMSEDSS > 3	SPMS	PPMS
25ft Walk (s)	4·8 (1·1)	6·7 (4)	15·3 (12)	11·9 (12)
SDMT (Number completed)	55·8 (12)	45·8 (13)	43·0 (9)	46·8 (10)
SDMT (Number correct)	54·9 (12)	45·4 (13)	42·0 (9)	46·0 (11)
9 Hole Peg – D (s)	18·9 (3)	24·3 (6)	35·0 (20)	29·8 (13)
9 Hole Peg – ND (s)	20·8 (3)	27·0 (13)	34·5 (11)	28·5 (9)

In the SPMS group, four patients could not complete the 25 ft walk as they are non-ambulatory. Two patients could not complete the 9 hole peg test due to deterioration of hand function.

D = dominant hand; ND = non-dominant hand.
